# Cytotoxicity and DNA Damage Ability of Isomeric *meso*-Tetra(cisplatin)porphyrins in Keratinocytes and Melanoma Cells Treated with Photodynamic Therapy

**DOI:** 10.1021/acsomega.5c08553

**Published:** 2026-03-25

**Authors:** Níckolas P. Peranzoni, Altevir R. Viana, Luana B. Trentin, Bernardo A. Iglesias, Erdi C. Aytar, André P. Schuch

**Affiliations:** a Laboratory of Photobiology, Department of Biochemistry and Molecular Biology, 28118 Federal University of Santa Maria−UFSM, Santa Maria, RS 97105-900, Brazil; b Laboratory of Bioinorganic and Porphyrinoid Materials, Department of Chemistry, Federal University of Santa Catarina−UFSC, Florianópolis, SC 88040-900, Brazil; c Faculty of Agriculture Department of Horticulture, 175652 Usak University, Uşak 64200, Türkiye

## Abstract

In this study, we evaluated and compared the cytotoxicity and DNA damage-inducing effects of two porphyrins conjugated with cisplatin at two different positions on the pyrrolic rings (**3-cis-PtTPyP** and **4-cis-PtTPyP**) with free cisplatin after exposure to white light. Both porphyrin molecules induced DNA damage and cytotoxic effects at lower concentrations (0.5–5 μM) when exposed to light. These molecules were more cytotoxic than free cisplatin to the human melanoma cell line exposed to white light since the observed IC_50_ values were 2.02 μM (**3-cis-PtTPyP**), 2.12 μM (**4-cis-PtTPyP**), and 13.25 μM (cisplatin). In addition, the results indicate that the treatment with these cisplatin-porphyrins followed by white light exposure was more cytotoxic to the melanoma cell line than to the keratinocyte cell line. Furthermore, both porphyrins presented higher DNA damage ability than free cisplatin, with **3-cis-PtTPyP** being the most genotoxic. It was also observed that both cisplatin-porphyrins significantly denatured egg albumin under light exposure, indicating a potential protein denaturation ability. Additionally, *in silico* analyses generated significant insights regarding the toxicological characteristics of both cisplatin-porphyrin compounds and their safety regarding clinical use. These findings demonstrate the effects of these cisplatin-conjugated porphyrins and highlight their differences from the conventional chemotherapeutic cisplatin.

## Graphical abstract

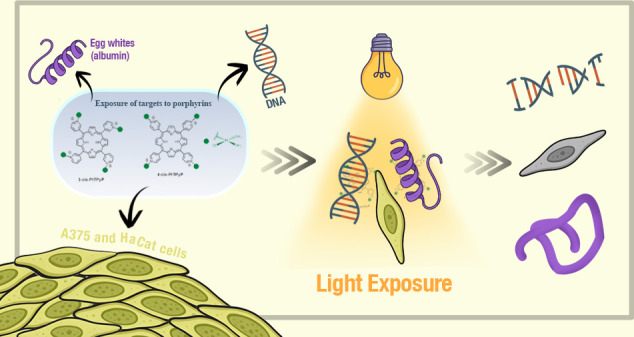

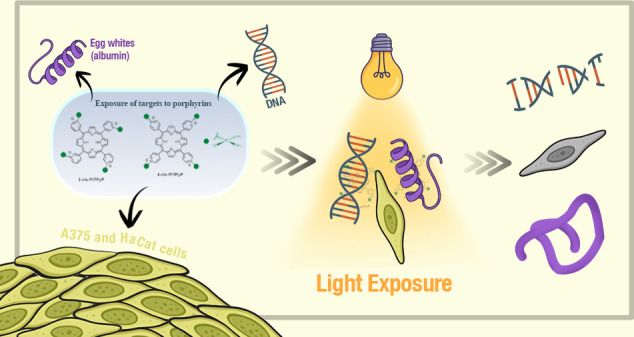

## 1 Introduction

Cancer remains one of the leading causes of death worldwide. Globally, the number of cases is expected to reach 35 million by 2050, demonstrating the need for continued development of new therapies for different types of cancer.^1,2^ Among them, skin cancer is one of the most common, including nonmelanoma types (basal cell carcinoma and squamous cell carcinoma) and melanoma, with the former ranking fifth among the most diagnosed types of cancer in 2020.^3^ Melanoma, on the other hand, is less frequently diagnosed but far more aggressive and metastatic, still accounting for the majority of deaths caused by skin cancer.^4,5^ Because of that, surgical resection and chemotherapy are among the preferred treatments for this disease. However, they are associated with many side effects and minimal survival benefits, which have led to the development and improvement of innovative, less invasive, and more precise therapies in the past decade, including therapies targeting BRAF and MEK (both proto-oncogenes), checkpoint immunotherapy, and photodynamic therapy (PDT).^6–8^

The use of light for disease treatment is not a new concept, and the first clinical application of this process was described by von Tappeiner and Jesionek in 1903 when they applied eosin topically to basal cell carcinomas (BCCs) followed by light exposure.^9^ This approach was later defined as “photodynamic therapy” (PDT), which encompasses the interaction of three main components to induce cell death: light, a photosensitizing agent (photosensitizer, PS), and molecular oxygen.^10^ Thus, PDT is a therapeutic approach for treating tumors and other illnesses, mainly skin diseases, based on the administration of a photosensitizing compound (called a photosensitizer, PS) at the target site followed by its activation by light with a wavelength range of optimal absorption by the PS. This process induces the generation of reactive oxygen species (ROS), leading to cellular damage.

For the photodynamic effect to occur, prior to light exposure, first the PS molecule needs to accumulate in the target site during a period defined as the “drug-light interval”, ensuring that the desired concentration is reached. Then, light is applied to the targeted tissue, resulting in the activation of the PS through the transition of ground-state electrons to an excited state, known as the triplet state, where the PS is more likely to interact with other molecules and cellular structures.^11,12^ Such a process can happen through two pathways: type I and type II. In the type I pathway, the triplet excited state of the photosensitizer (PS) can interact directly with a substrate through electron transfer or hydrogen abstraction reactions, causing immediate damage to the cellular membrane, proteins, DNA, and other molecules. Alternatively, it can produce highly reactive free radicals and radical ions that can further react with molecular oxygen (O_2_) to generate ROS, such as superoxide anions (O_2_^•–^), hydrogen peroxide (H_2_O_2_), and hydroxyl radicals (•OH).^13^ The ROS can oxidize cellular structures, subsequently leading to cell death.^14^ In contrast, in the type II pathway, the interaction between the excited photosensitizer with molecular oxygen occurs through direct energy transfer, resulting in the formation of singlet oxygen (^1^O_2_) and the return of the PS to its ground state.^13^

There are three main characteristics that must be present in a molecule to be considered a suitable PS for PDT. First, a good quantum yield of ROS is needed to enhance the effects of the treatment.^14,15^ The second essential attribute is the induction of damage only when the molecule is excited by light.^16,17^ Finally, an ideal PS should be cost-effective and readily available. Porphyrins are a group of organic molecules widely used in PDT. These molecules are characterized by their free-based tetrapyrrolic macrocyclic structure and significant modular potential, which have been tested for their differential cytotoxicity over the years. One of the main attributes of these molecules is their ability to absorb light across a broad spectrum, ranging from the UV region to near-infrared (NIR), which, in addition to their ability to associate with other compounds, makes porphyrins ideal PSs for PDT.^18,19^ Furthermore, the solubility and interactions of molecular ligands with the porphyrin can be easily modified to enhance PDT effects.^20–22^ These specific modifications can be made by adding a metal atom at the center of the porphyrin macrocycle^23,24^ or by incorporating entire molecules, such as chemotherapeutic agents, as external groups.

Cisplatin is a first-generation platinum-based chemotherapeutic drug, first synthesized by Michele Peyrone in 1845, whose anticancer properties were only discovered at the end of the 1960s when Dr. Barnett Rosenberg identified its efficacy against sarcoma 180 and leukemia L1210.^25^ Since then, this compound has been used to treat a variety of cancers, such as ovarian cancers with great efficacy and lung, cervical, bladder, lymphoma, head and neck, and prostate cancers, among others, leading to its approval by the FDA in 1978.^26^ In general, cisplatin binds covalently to DNA bases, forming cross-links and intra- and interstrand DNA adducts, which block DNA replication and transcription, activating a series of transduction pathways that ultimately lead to cell death by necrosis or apoptosis.^27–29^ Despite its efficacy, chemotherapeutic treatment with cisplatin poses several challenges, including its association with various systemic toxicities, most notably nephrotoxicity. This occurs due to the lack of selectivity exhibited by the molecule, leading to damage in nontarget tissues, such as the kidneys.^30^ Additionally, mechanisms of cisplatin resistance are diverse, including reduced intracellular accumulation of cisplatin in cancer cells and increased sequestration of the compound by redox system molecules with nucleophilic properties, such as glutathione (GSH) and metallothioneins.^31^ Therefore, strategies to overcome these challenges are essential to enhancing the effectiveness of cisplatin treatment.

In this sense, the combination of porphyrins and cisplatin and their use in PDT against tumor cells resistant and sensitive to cisplatin have already been previously described.^32^ Therefore, this study aims to explore the DNA damage ability and cytotoxic effects of two novel cisplatin-porphyrin conjugates against cancerous and nonmalignant skin cells. By combining the phototoxic properties of porphyrins with the chemotherapeutic potential of cisplatin, this approach seeks to enhance treatment efficacy while minimizing systemic toxicity, offering new perspectives for the development of light-activated anticancer therapies.

## 2 Methods

### 2.1 Porphyrins and Cells

Two free-base tetra-cationic porphyrins were used, each containing four cisplatin molecules linked to a pyridine ring at the *meso* positions of the porphyrin, altering the position of the nitrogen atom in the rings for each porphyrin (Figure 1). Also, the synthesis and characterization of these derivatives have been previously described by Naik and co-workers.^32^ The UV–vis absorption spectra and the electrochemical analysis by cyclic voltammetry of free cisplatin, **3-cis-PtTPyP**, and **4-cis-PtTPyP** are provided in the Supporting Information (Figures S1 and S3, respectively).

**Figure 1:**
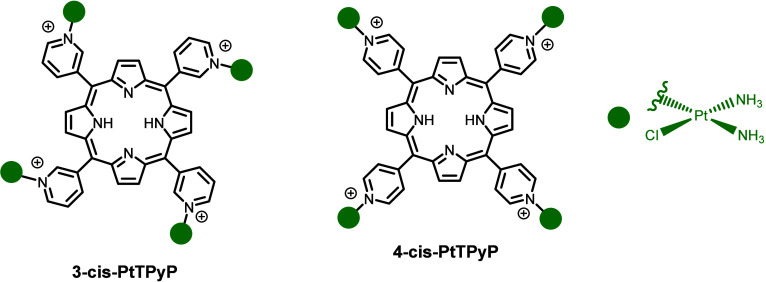
Representative chemical structure of isomeric *meso*-tetra-cationic porphyrin derivatives with peripheral cisplatin and free cisplatin. Nitrate (NO_3_^–^) counterions are omitted for more clarity.

The cell lines used were A375 (human melanoma) and HaCat (nonmalignant human keratinocytes), both obtained from the Cell Bank of Rio de Janeiro (BCRJ). Cells were grown in DMEM (Dulbecco’s modified Eagle’s medium) supplemented with 10% FBS and 1% antibiotics (100 U/mL penicillin and 100 U/mL streptomycin). The culture flasks (25 cm^2^) were maintained in an incubator at 37 °C with 5% CO_2_. For the MTT and DCFH-DA assays, 24 h prior the beginning of treatments, 1 × 10^4^ cells were plated per well of 96-multiwell plates in a final volume of 200 μL of DMEM. For the colony formation assay, 500 cells were plated in six-well plates in a final volume of 2 mL of DMEM. For treatments, DMEM was removed, and cells were incubated with porphyrins (or free cisplatin) diluted in PBS 1× for a period of 90 min during the light exposure or kept in the dark. After treatment, PBS buffer containing porphyrins or free cisplatin was replaced by DMEM supplemented with 10% FBS and 1% antibiotics and maintained in an incubator at 37 °C with 5% CO_2_ for the subsequent analyzes.

### 2.2 White Light Exposure of Biological Samples

Human cell lines mentioned above and plasmid DNA samples were exposed to light in a homemade white light LED array system (Philips LEDs, irradiance of 50 mW/cm^2^). This light source has an emission range between 380 and 780 nm, with a more intense emission peak in the blue region. The human cells were exposed to light inside 96-multiwell plates in PBS 1×, and DNA samples were exposed and diluted in Tris-HCL (pH 8,0) using a DNA dosimeter system.^33^ The exposures were performed at a distance of 5 cm from the light source for a 90 min period, reaching the dose of 27 J/cm^2^. The irradiance of this LED array system was measured with the GSP-1B gonio-spectrophotometer (Avian Technologies, USA), and the emission spectrum is provided in the Supporting Information (Figure S2).

### 2.3 Quantification of DNA Damage

To detect and quantify the DNA damage induced by light exposure, the plasmid DNA pCMUT (1.768 kbp, C: chloramphenicol resistance; MUT: *supF* gene, a target for mutagenesis studies) samples were exposed to white light in the presence of different concentrations of both porphyrins and free cisplatin (0.1, 0.5, 1.0, and 5.0 μM). Immediately after exposure, the DNA was incubated at 37 °C for 1 h with the enzymes formamidopyrimidine-DNA glycosylase (Fpg, which recognizes and cleaves oxidized purines) and endonuclease III (Endo III, which recognizes and cleaves oxidized pyrimidines). Additionally, the DNA was also incubated at the same temperature without the presence of enzymes to visualize single-strand breaks (SSBs) or double-strand breaks (DSBs). These samples were subjected to electrophoresis on a 0.8% agarose gel. The documentation of this gel was carried out using the Amersham Imager 600 (General Electric). In the absence of damage, the plasmid DNA remains in its supercoiled form (FI), producing the lower band. In the case of damage formation, the DNA appears in its circular, more relaxed form (FII), producing the upper band in the agarose gel. The quantification of the damage was performed by densitometry of the bands using the Image Quant 300 program (GE Healthcare, USA).^33^

### 2.4 ROS Production

To quantify the formation of reactive species in the cells, a fluorimetric assay was performed using 2,7-dichlorofluorescein diacetate (DCFH-DA). The assay consisted of placing 10 μL of DCFH-DA solution (0.1 mM) on the cells before white light exposure. After 90 min of light exposure treatment, 50 μL of the supernatant from the treated cells was transferred to wells of a 96-well black plate (protected from light) containing 65 μL of Tris-HCl. The results were read using a spectrofluorometer with emission and excitation wavelengths of 525 and 488 nm, respectively. A 100 μM hydrogen peroxide solution was added to the cells and used as a positive control.^34^

### 2.5 Cytotoxicity

The cytotoxicity of the compounds was investigated using the MTT assay performed 24 h after 90 min of light exposure treatment. Prior to the treatment, the culture medium was removed, 150 μL of PBS 1× containing free cisplatin or each cisplatin-porphyrin concentration was added to each well, and the cells were exposed to a white light LED irradiation source as described above. In parallel, the same treatment was performed but without cell exposure to the white light source (dark condition). Immediately after light exposure (or dark condition), the solution containing porphyrin was removed, and cells were washed with PBS (1×). Then, 200 μL of DMEM was added, and the cells were maintained in an incubator at 37 °C with 5% CO_2_ for 24 h. After a 24 h incubation period, DMEM was removed, cells were washed with PBS (1×), 20 μL of the MTT solution (5.0 mg/mL) was added to each well, and the microplates were returned to 37 °C and 5.0% CO_2_ atmosphere for 4 h. Subsequently, supernatant was collected, and formazan crystals resulting from the reduction of MTT by cellular succinate dehydrogenase activity were solubilized in 200 μL of DMSO. Then, absorbance was measured at 570 nm using a microplate reader (SpectraMax i3x, Molecular Devices). The negative control was considered with 100% of cell viability for comparison with free cisplatin- or porphyrins-treated samples. A 100 μM hydrogen peroxide solution was added to the cells and used as a positive control.

### 2.6 Colony Formation

For the colony formation assay, 500 cells were seeded in six-well plates and allowed to adhere to the plastic surface for 24 h in an incubator at 37 °C with 5% CO_2_. On the following day, the cells were treated with the respective IC_50_ concentration of cisplatin-porphyrins or free cisplatin (see Table 1) diluted in PBS (1×) and exposed for 90 min to white light, while the other half of the plates were kept protected from light for the same period. Subsequently, the supernatant was removed, fresh culture medium was added, and the cells were returned to 37 °C under a 5.0% CO_2_ atmosphere for 10 days. Colony formation was visualized and quantified after staining with a 10% crystal violet solution for 30 min at 37 °C.^35^

**Table 1:** IC_50_ Values of **3-cis-PtTPyP**, **4-cis-PtTPyP**, and Free Cisplatin against A375 (Melanoma) and HaCaT (Keratinocyte) Cell Lines

treatments	A375 (μM)	HaCat (μM)
cisplatin (dark)	9.13	7.70
cisplatin (light)	13.25	8.13
3-cis-PtTPyP (dark)	4.97	5.74
**3-cis-PtTPyP** (light)	2.02	2.26
**4-cis-PtTPyP** (dark)	4.45	5.00
**4-cis-PtTPyP** (light)	2.12	2.38

### 2.7 Protein Denaturation Potential

To assess the protein damage induced by free cisplatin or cisplatin-porphyrins, an assay based on the denaturation of egg white albumin was performed, as the solubility of this protein is very similar to that of proteins found in the epidermis.^36^ A total of 2.0 mL of egg white was added to solutions containing 0.1 and 5.0 μM porphyrin or free cisplatin. The samples were incubated under white light and dark conditions as previously described to determine the effects on protein denaturation. After the irradiation period, samples’ transmittance was measured at 660 nm using a UV–vis spectrophotometer, a wavelength commonly used to assess solution turbidity, which indicates the degree of albumin denaturation.^37,38^ The experiments were performed in triplicate, and the results were expressed as the mean ± standard deviation of the samples’ transmittance.

### 2.7. *In* *Silico* Toxicity Profile

The toxicity profiles of the *meso*-tetra­(cisplatin)­porphyrins used in this work were assessed using the pkCSM pharmacokinetics software (https://biosig.lab.uq.edu.au/pkcsm/, on October 3, 2024). It provides analyses of the compound pharmacokinetic profiles (ADME) and toxicity, thereby suggesting safety indicators based on their toxicity profiles obtained *in silico*. The evaluated parameters included LD50 (mg/kg), toxicity class, hepatotoxicity, neurotoxicity, nephrotoxicity, respiratory toxicity, blood–brain barrier (BBB) permeability, ecotoxicity, clinical toxicity, nutritional toxicity, and activation of cytochrome CYP enzymes (CYP1A2, CYP2C19, CYP2C9, CYP2D6, CYP3A4, and CYP2E1). These parameters were analyzed to understand the safety and potential interactions of *meso*-tetra­(cisplatin)­porphyrins used in this work to induce side effects.

### 2.9 Statistical Analysis

All experiments were performed in triplicate and independently repeated at least three times. The results were analyzed using GraphPad Prism (version 8.0.1) through a one-way ANOVA followed by Tukey’s *post hoc* test, with statistical significance set at *p* < 0.05.

## 3 Results and Discussion

The experiments performed with **3-cis-PtTPyP** and **4-cis-PtTPyP** allowed us to directly test how the positional isomerism of cisplatin influenced the biological effects of these cisplatin-porphyrin conjugates under photodynamic activation. This chemical variation was systematically correlated with multiple biological end points, including DNA damage induction, reactive oxygen species production, cytotoxicity in both tumor and nonmalignant skin cell lines, and protein denaturation. By aligning precise molecular design with targeted biological assays, we were able to elucidate structure–activity relationships that could not be predicted from chemical properties alone, providing mechanistic insights relevant to the rational development of dual-action PDT agents.

### 3.1 DNA Damage Ability

Through the plasmid DNA photocleavage assay, it was possible to identify and quantify DNA lesions induced by *meso*-tetra­(cisplatin)­porphyrins and free cisplatin (Figure 2A). Both cisplatin-porphyrins, when exposed to white light irradiation at the highest tested concentration, caused significant DNA base oxidation, particularly in purines, as evidenced by the activity of the Fpg enzyme. The porphyrin **3-cis-PtTPyP** exhibited the most pronounced damage activity, reaching approximately 0.6 breaks per kbp (Figure 2B). Similarly, **4-cis-PtTPyP** displayed a comparable damage pattern, with up to 0.4 breaks per kbp at a concentration of 5.0 μM (Figure 2C). In contrast, free cisplatin induced a significantly lower number of DNA breaks, with a maximum of 0.14 breaks per kbp (Figure 2D). Notably, in the absence of light, none of the tested compounds induced significant DNA damage, reinforcing the need of light activation for DNA base oxidation to occur (Figure 2E–G).

**Figure 2:**
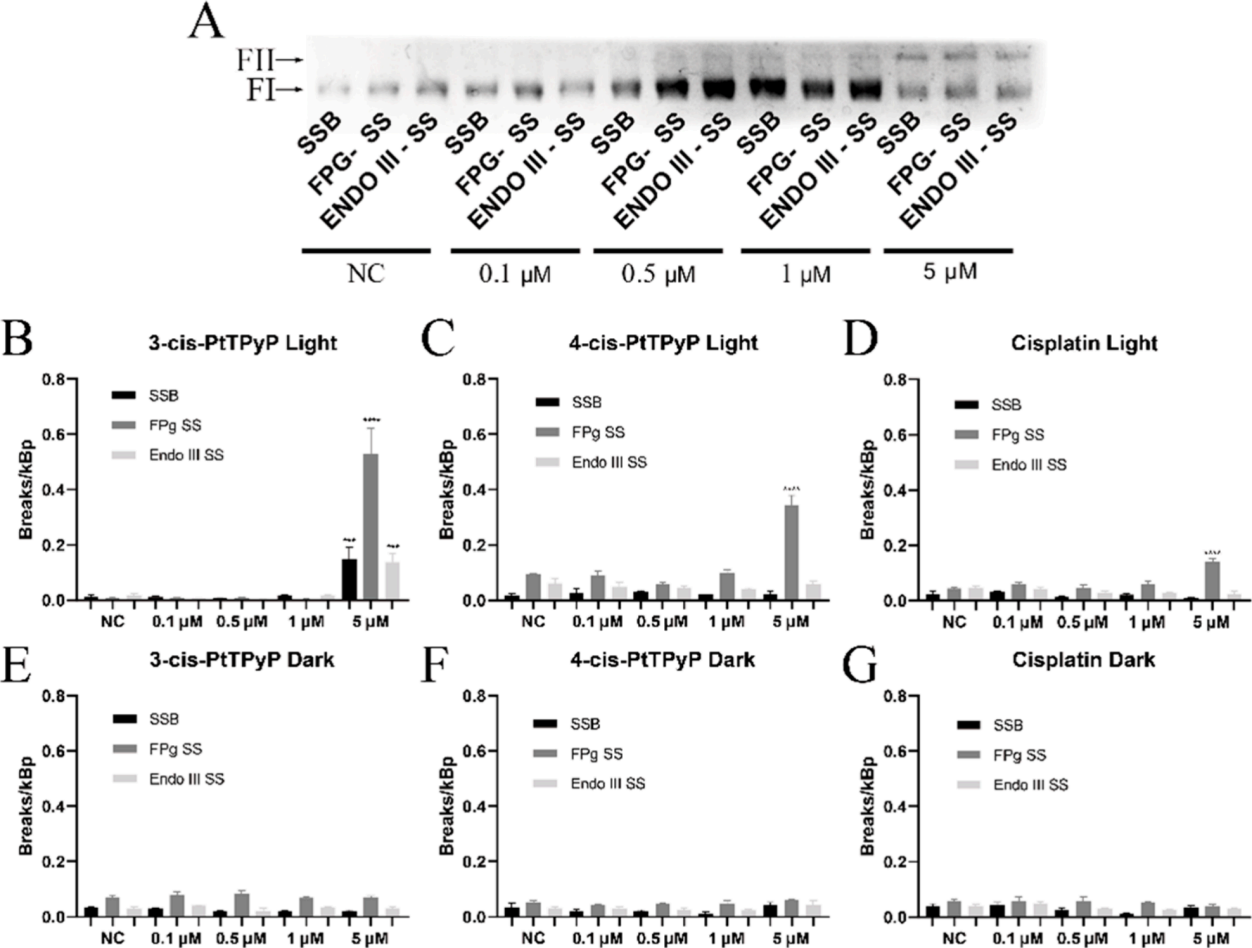
Quantification of DNA damage induced by **3-cis-PtTPyP**, **4-cis-PtTPyP**, and free cisplatin under white light (irradiance of 50 mW/cm^2^and light dosage of 270 J/cm^2^at 90 min) and dark condition. (A) Representative electrophoresis gel showing plasmid DNA migration after treatment with different concentrations of the compounds followed by incubation with Fpg (Fpg-sensitive sites; Fpg-SS) and Endo III (Endo III-sensitive sites; Endo III-SS) enzymes, or without enzymes (SSBs; single-strand breaks), after visible light exposure. (B–D) Quantification of DNA damage caused by **3-cis-PtTPyP**, **4-cis-PtTPyP**, and cisplatin under light conditions. (E–G) Quantification of DNA damage in dark conditions. NC: negative control (nontreated DNA sample). Data represent the mean and standard deviation of three independent experiments. ****p < 0.0001.

The obtained results indicate a higher induction of oxidized bases by cisplatin-porphyrins exposed to light compared with the exposure of free cisplatin. This difference is consistent with the mechanisms of action of PS used in PDT. The higher number of oxidized purines in the light-treated DNA samples in the presence of porphyrins, as evidenced by the Fpg-SS, suggests the formation of singlet oxygen, which preferentially reacts with guanine.^39,40^ Unfortunately, we could not evaluate here the DNA base oxidation mechanism by the addition of ROS scavengers in this assay. However, a previous published work that used the same cisplatin-porphyrins used here demonstrated that, indeed, singlet oxygen, and probably hydroxyl radical, can be formed under white light exposure.^41^ In another similar work, porphyrin containing ruthenium, which is a transition metal like cisplatin, demonstrated significant potential in inducing DNA damage when exposed to light, reaching approximately 2.5 breaks per kbp at 5.0 μM. Curiously, the amount of DNA lesions was not attenuated by the addition of ROS scavengers, suggesting the existence of an additional mechanism for DNA break induction.^42^ Further experiments are also needed to evaluate if these both cisplatin-porphyrin conjugates can induce other types of DNA damage, such as DNA cross-links, which are a well-documented DNA lesion induced by free cisplatin.^43–45^

### 3.2 Cytotoxicity

Both *meso*-tetra­(cisplatin)­porphyrins demonstrated cytotoxic activity 24 h after white light exposure (Figure 3). After light treatment, **3-cis-PtTPyP** exhibited significant cytotoxic activity at all concentrations in the A375 human melanoma cell line, while the porphyrin **4-cis-PtTPyP** caused significant cytotoxicity only at higher concentrations of 1 and 5 μM (Figure 3A). However, in the nonmalignant HaCat cells, only concentrations equal to or higher than 0.5 μM of the **3-cis-PtTPyP** were significantly cytotoxic. The **4-cis-PtTPyP** also caused significant cytotoxicity at the higher concentrations of 1 and 5 μM (Figure 3B). In the dark, only the concentration of 5 μM of **3-cis-PtTPyP**, as well as 1 and 5 μM of **4-cis-PtTPyP,** was toxic to A375 cells (Figure 3C). In the HaCat cells, only 5 μM of both **3-cis-PtTPyP** and **4-cis-PtTPyP** was cytotoxic (Figure 3D). The values of IC_50_ induced by cisplatin, **3-cis-PtTPyP**, and **4-cis-PtTPyP** in each cell line were also calculated. The **3-cis-PtTPyP** was the most cytotoxic compound to the human melanoma A375 cell line (Table 1).

**Figure 3:**
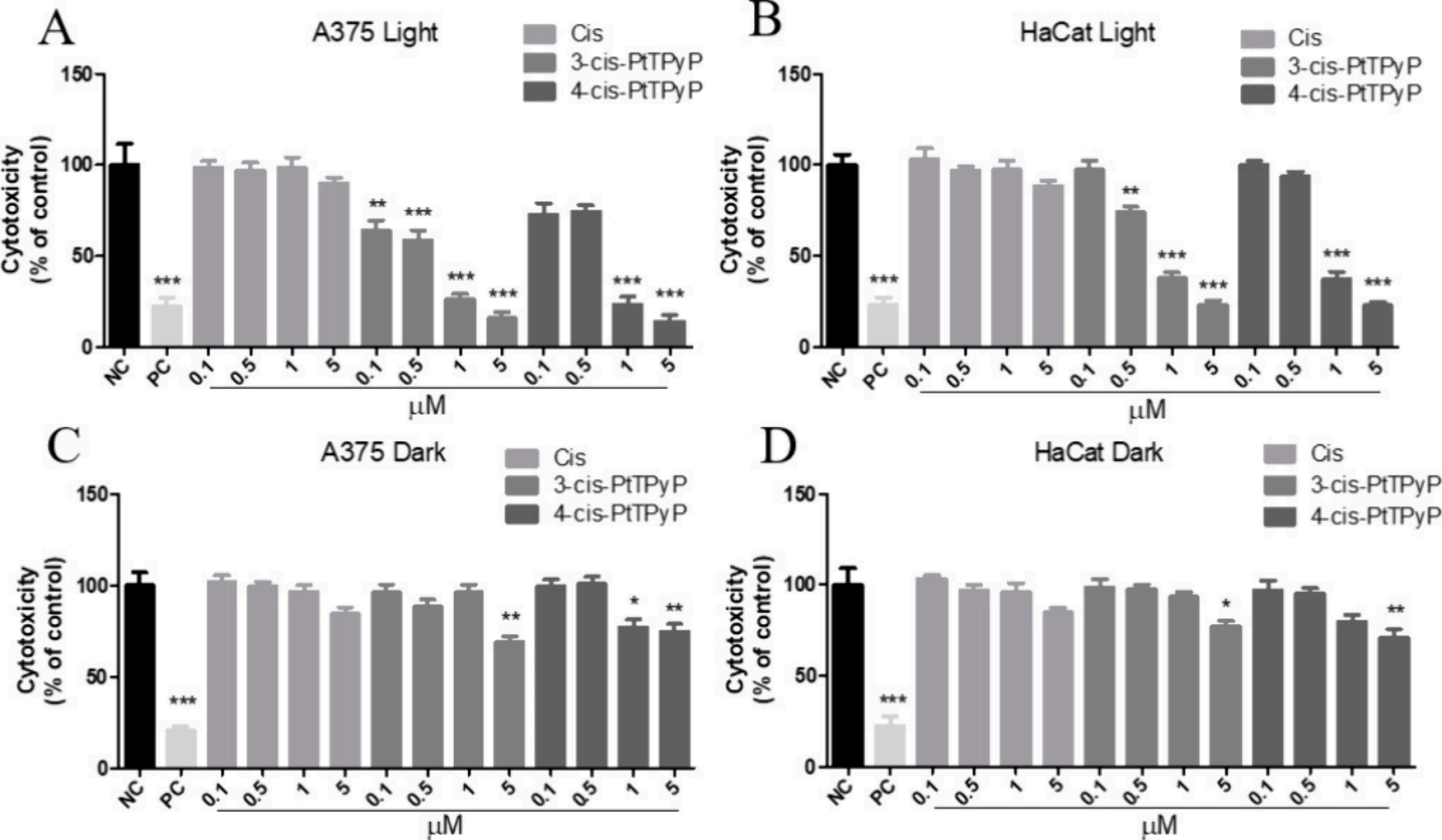
Cytotoxicity (MTT assay) 24 h after treatment of A375 (human melanoma) and HaCat (human keratinocytes) cells with different concentrations (0.1–5.0 μM) of **3-cis-PtTPyP**, **4-cis-PtTPyP**, and free cisplatin. (A) A375 cells under light exposure, (B) HaCat cells under light exposure, (C) A375 cells in the dark, and (D) HaCat cells in the dark. Data represent the mean and standard deviation of three independent experiments performed in triplicate. *p* < 0.01 (**), *p* < 0.001 (***), *p* < 0.0001 (****). NC: negative control; cells in DMEM. PC: positive control; hydrogen peroxide 100 mM.

The results of the colony formation assay performed 10 days after treatments indicate that the A375 melanoma cell line is more sensitive than the HaCat to free cisplatin and both cisplatin-porphyrins whether in the light or in the dark conditions (Figure 4). Light exposure treatment drastically reduced cell survival of both cell lines, indicating that the light activation mechanism of both cisplatin-porphyrins is responsible for the observed cell death. On the other hand, both cisplatin-porphyrins were toxic in the dark only for the tumor cell line A375, decreasing the number of cell colonies to about 60% in relation to the nontreated control (Figure 4A). The cisplatin-porphyrins were not toxic to the HaCat cell line in the dark (Figure 4B).

**Figure 4:**
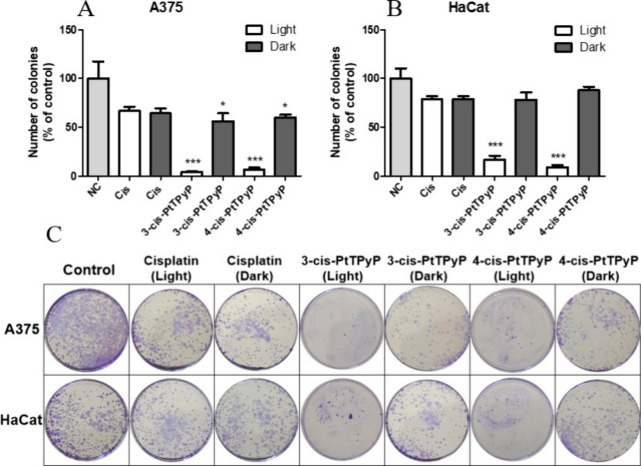
Colony formation assay performed 10 days after treatments of A375 (human melanoma) and HaCat (human keratinocytes) cells with the IC_50_ of **3-cis-PtTPyP**, **4-cis-PtTPyP**, and free cisplatin. (A) Percentage (%) of colonies of A375 cells in relation to the nontreated control. (B) Percentage (%) of colonies of HaCat cells in relation to the nontreated control. (C) Representative images of colony formation 10 days after each treatment of A375 and HaCat cells. Data represent the mean and standard deviation of three independent experiments performed in triplicate. *p* < 0.05 (*), *p* < 0.001 (***), NC: negative control.

Furthermore, the fluorometric assay (DCFH-DA) indicates that the porphyrin **3-cis-PtTPyP** led to a significant production of ROS at concentrations of 1.0 and 5.0 μM in the A375 tumor cells when exposed to white light (Figure 5A), although the porphyrin **4-cis-PtTPyP** led to a significant production of ROS at concentrations of 0.5, 1.0, and 5.0 μM (Figure 5A). On the other hand, **3-cis-PtTPyP** induced a significant production of ROS at 5.0 μM in HaCat cells, while **4-cis-PtTPyP** induced a significant amount of ROS at 1.0 and 5.0 μM in this cell line (Figure 5B). None of the treatments resulted in ROS production in the dark, suggesting that ROS generation was dependent on light exposure and the porphyrin concentration (Figure 5C,D).

**Figure 5:**
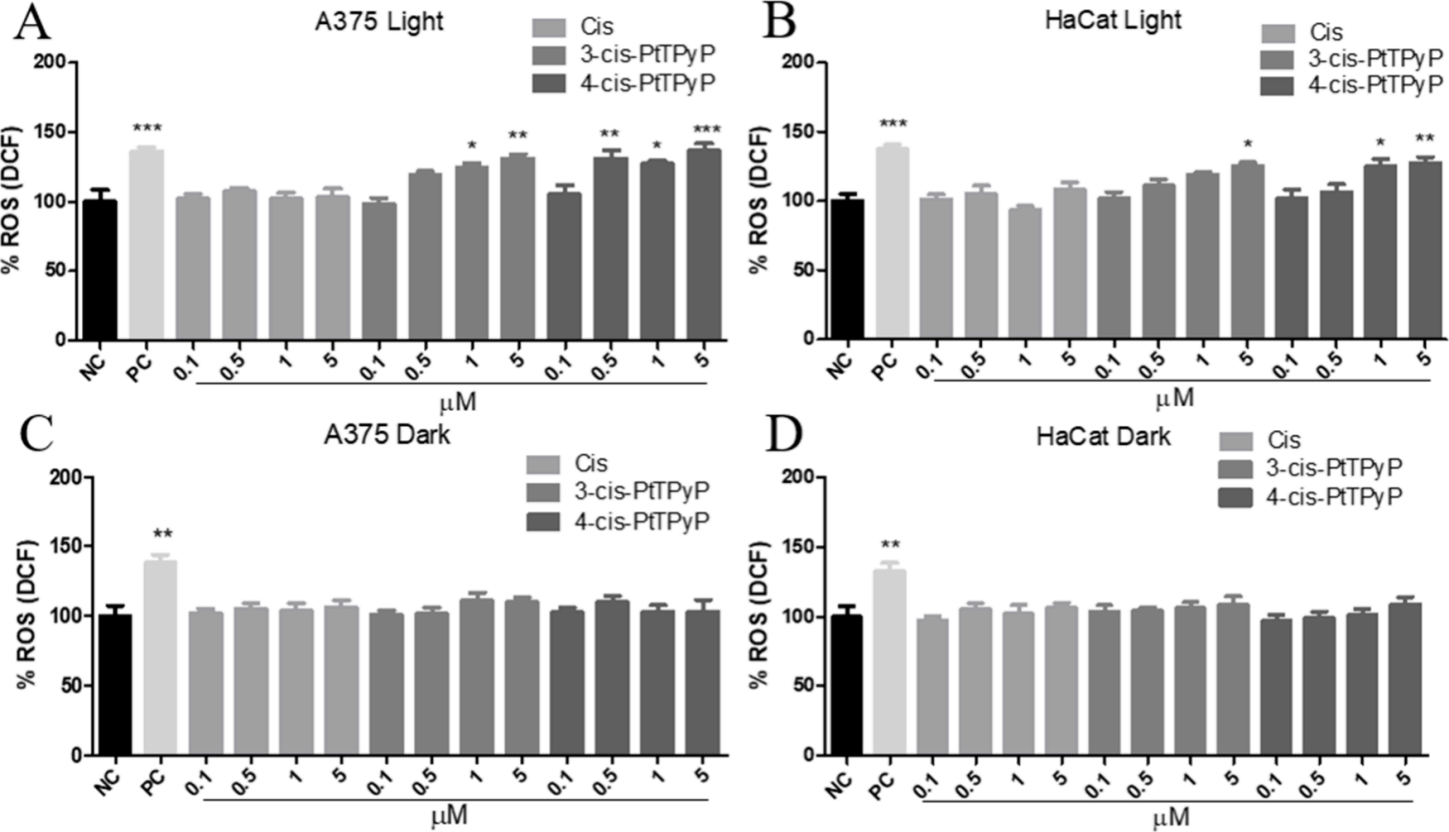
Reactive oxygen species (ROS) production (DCFH-DA assay) immediately after white light treatment of A375 (melanoma) and HaCat (human keratinocytes) cells with different concentrations (0.1–5.0 μM) of **3-cis-PtTPyP**, **4-cis-PtTPyP**, and free cisplatin. (A) A375 cells under light exposure, (B) HaCat cells under light exposure, (C) A375 cells in the dark, and (D) HaCat cells in the dark. Data represent the mean and standard deviation of three independent experiments performed in triplicate. *p* < 0.05 (*), *p* < 0.01 (**), *p* < 0.001 (***). NC: negative control; cells in DMEM. PC: positive control; hydrogen peroxide 100 mM.

The cytotoxicity induced by 1.0 and 5.0 μM of both *meso*-tetra­(cisplatin)­porphyrins in A375 tumor cells was much higher in comparison to free cisplatin after exposure to white light (Figures 3 and 4 and Table 1). However, **3-cis-PtTPyP** at 0.1 and 0.5 μM was also significantly more cytotoxic than free cisplatin (Figure 3A). In the HaCat cells, 0.5 μM of **3-cis-PtTPyP** was also significantly more cytotoxic than free cisplatin (Figure 3B). The clonogenic assay performed 10 days after treatments also confirms that both cisplatin-porphyrins are more cytotoxic than free cisplatin to both cell lines, with the A375 cell line being more sensitive than HaCat (Figure 4). These results highlight the higher selectivity of A375 tumor cells for both porphyrins in comparison to free cisplatin, as well as in comparison to HaCat cells. In fact, malignant cells generally show upregulation of low-density lipoprotein (LDL) receptors (e.g., apo B/E receptor), accounting for the preferential labeling of malignant cells by some porphyrins.^46^ Therefore, it is thought that these cisplatin-porphyrins’ cytotoxicity observed in A375 tumor cells could result from their delivery via the LDL receptor pathway. Thus, this result suggests a potential reduction in the systemic side effects common to conventional chemotherapeutic agents such as cisplatin. Moreover, the dependence on light exposure may provide spatial and temporal control of toxicity, minimizing damage to nonmalignant tissues.^47,48^ Similarly, *meso*-tetra­(pyridyl)­porphyrins with peripheral palladium­(II) bipyridyl complexes showed a comparable effect, reducing A375 cell viability by approximately 65% at a concentration of 0.56 μM, a percentage that remained unchanged even at higher concentrations of up to 10.5 μM, although also causing significant damage to nonmalignant cells (L929 cell line).^49^

The results also suggest that the production of ROS may have a major role in the cytotoxicity induced by these cisplatin-porphyrins, mainly in the highest concentrations here evaluated. However, despite the light-induced ROS formation being more efficient in the treatments with **4-cis-PtTPyP** than **3-cis-PtTPyP** (Figure 5), the amount of induced oxidized DNA damage was higher in the light treatment with **3-cis-PtTPyP** (Figure 3). Therefore, further studies are necessary to demonstrate other possible mechanisms that can be associated with porphyrins-induced cytotoxicity, such as the evaluation of formation of DNA cross-links, which are characteristic DNA lesions formed by cisplatin. This type of cisplatin-DNA adducts interferes with DNA replication and transcription, potentially leading to cell death through different signaling pathways.^50^ Furthermore, the interaction of porphyrins with cellular organelles may play a key role in inducing cell death and also deserves investigation.

### 3.3 Protein Denaturation Potential

The protein denaturation assay allowed for the evaluation of the interaction between *meso*-tetra­(cisplatin)­porphyrins and free cisplatin with egg proteins, specifically albumin. Transmittance analysis revealed that both porphyrins (**3-cis-PtTPyP** and **4-cis-PtTPyP**) induced a significant reduction in the transmittance of the solution at both tested concentrations (0.1 and 5.0 μM) but exclusively under light exposure (Figure 6). This finding suggests that the excitation of molecules by white light is essential for their interaction with albumin, promoting its denaturation and consequently increasing the turbidity of the solution. Among the tested compounds, **4-cis-PtTPyP** at a concentration of 5.0 μM demonstrated the most pronounced effect, reducing the transmittance by 54%. In contrast, free cisplatin, regardless of the concentration used, did not promote significant albumin denaturation, suggesting a lower interaction with the protein in this experimental model.

**Figure 6:**
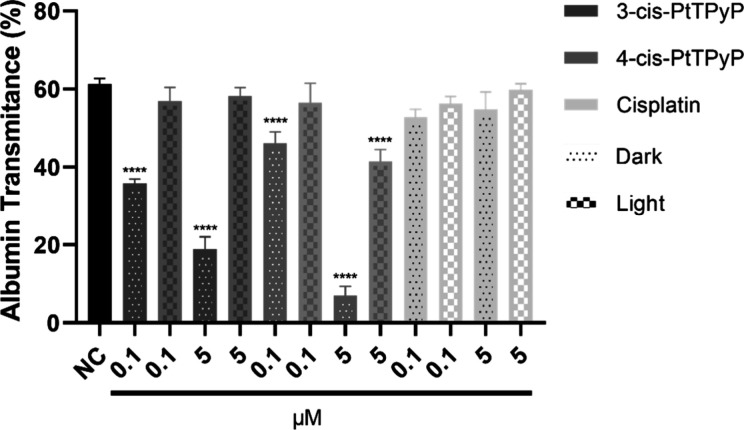
Assessment of protein denaturation potential of **3-cis-PtTPyP**, **4-cis-PtTPyP**, and free cisplatin through albumin denaturation. Albumin transmittance at 660 nm was measured after incubation with 0.1 and 5.0 μM of each compound under white light (irradiance of 50 mW/cm^2^ and light dosage of 270 J/cm^2^ at 90 min) and dark conditions. A decrease in transmittance indicates protein denaturation. Data represent the mean and standard deviation of three independent experiments. ****p < 0.0001. NC: negative control; egg whites and water.

The observation that albumin denaturation occurs only when cisplatin-porphyrins are excited by light suggests that the protein damage induced is mediated by a photodynamic process. This phenomenon indicates a possible interaction between singlet oxygen and proteins, promoting the oxidation of amino acids and resulting in conformational changes that lead to albumin denaturation. This mechanism is characteristic of a type II pathway, in which ROS, such as ^1^O_2_, play a central role in the structural modification of biomolecules.^51^ However, amino acid oxidation can also occur through the type I mechanism, in which electron transfer induces direct protein damage. This process has already been described in the literature for human serum albumin, whose tryptophan residues can interact with singlet oxygen and additionally suffer damage via electron transfer.^52^ These processes can lead to the accumulation of denatured proteins within the cell, triggering stress responses such as the heat shock protein pathway^53^ and mechanisms of apoptosis and autophagy.^54^

### 3.4 *In* *Silico* Profile

To evaluate the safety of the *meso*-tetra­(cisplatin)­porphyrins for further perspectives regarding clinical use, we determined the ADMET and toxicity profiles of **3-cis-PtTPyP** and **4-cis-PtTPyP** (Table 2). Both compounds exhibit no Ames toxicity, indicating that they do not possess mutagenic properties. The maximum tolerated dose is 0.229 log mg/kg/day for **3-cis-PtTPyP** and 0.026 log mg/kg/day for **4-cis-PtTPyP**. Neither compound acts as a hERG I inhibitor, while **4-cis-PtTPyP** is identified as a hERG II inhibitor, suggesting potential cardiotoxicity. The acute oral toxicity (LD50) values are recorded at 2.508 mol/kg for **3-cis-PtTPyP** and 2.671 mol/kg for **4-cis-PtTPyP**. The lowest observable adverse effect level (LOAEL) for chronic toxicity is −0.044 log mg/kg_bw/day for **3-cis-PtTPyP** and −0.139 log mg/kg_bw/day for **4-cis-PtTPyP**. No hepatotoxicity or skin sensitization is observed for either compound. The toxicity to *Tetrahymena pyriformis* is measured at 0.285 log ug/L for both compounds, while the minnow toxicity is 5.216 log mM for **3-cis-PtTPyP** and 4.253 log mM for **4-cis-PtTPyP**. These findings provide significant insights into the toxicological characteristics of both compounds and their safety for further clinical applications. However, it is important to mention that further studies are needed to confirm the real toxicity of these molecules *in vivo.*

**Table 2:** ADMET (Absorption, Distribution, Metabolism, Excretion, and Toxicity) Profile

properties	3-cis-PtTPyP	4-cis-PtTPyP
Ames^a^ toxicity	no	no
max. tolerated dose (human) (log mg/kg/day)	0.229	0.026
hERG I inhibitor	no	no
hERG II inhibitor	no	yes
oral rat acute toxicity (LD50)(mol/kg)	2.508	2.671
oral rat chronic toxicity (LOAEL) (log mg/kg_bw/day)	–0.044	–0.139
hepatotoxicity	no	no
skin sensitization	no	no
*T. pyriformis* toxicity (log μg/L)	0.285	0.285
minnow toxicity (log mM)	5.216	4.253

a AMES: mutagenic test.

The photodynamic effects induced by **3-cis-PtTPyP** and **4-cis-PtTPyP** together with the ADMET profiles provide significant insights regarding their therapeutic potential and toxicity risks. First, the results from the AMES toxicity test indicate that neither compound is mutagenic, which suggests a low risk of genetic mutations during photodynamic therapy. However, **4-cis-PtTPyP** acts as a hERG II inhibitor, indicating potential cardiotoxicity, whereas **3-cis-PtTPyP** does not inhibit hERG channels, making it a safer alternative concerning cardiovascular side effects. In terms of oral toxicity, both compounds exhibit similar LD_50_ values, indicating comparable acute oral toxicity levels. However, **4-cis-PtTPyP** has a lower LOAEL (lowest observed adverse effect level) value, suggesting that chronic toxic effects may occur at lower doses with prolonged use (Table 2). Both compounds lack hepatotoxic effects, which are particularly promising for **3-cis-PtTPyP**, as it can apparently be safely used. Nonetheless, the higher minnow toxicity observed for **4-cis-PtTPyP** indicates a higher environmental toxicity risk that warrants consideration.

Table 3 also provides interesting data regarding different toxicity models. The acute toxicity profile of these compounds reveals LD_50_ values of 501 mg/kg for **3-cis-PtTPyP** and 493 mg/kg for **4-cis-PtTPyP**, classifying both in toxicity class 4, indicating low toxicity. Both compounds exhibit neurotoxicity and respiratory toxicity, while they are inactive with regard to hepatotoxicity and nephrotoxicity. Additionally, they can cross the blood–brain barrier and show clinical toxicity potential. Regarding cytochrome P450 enzymes, both compounds are active on CYP2D6 but are inactive on other cytochromes. In conclusion, both porphyrins demonstrate potential as candidates for photodynamic therapy, with **3-cis-PtTPyP** emerging as a particularly strong candidate due to its higher cytotoxicity in melanoma cells and DNA damage induction, as well as cardiovascular and hepatotoxic safety. However, its high toxicity toward nonmalignant cells at elevated doses requires careful adjustments. On the other hand, **4-cis-PtTPyP** shows potential cardiac risks due to its hERG inhibition, necessitating close monitoring of cardiovascular effects during the development of clinical studies.

**Table 3:** Acute Toxicity Profile

toxicity model	3-cis-PtTPyP	4-cis-PtTPyP
LD_50_(mg/kg)	501	493
toxicity class	4	4
hepatotoxicity	inactive	inactive
neurotoxicity	active	active
nephrotoxicity	inactive	inactive
respiratory toxicity	active	active
BBB barrier	active	active
ecotoxicity	inactive	inactive
clinical toxicity	active	active
nutritional toxicity	inactive	inactive
cytochrome CYP1A2	inactive	inactive
cytochrome CYP2C19	inactive	inactive
cytochrome CYP2C9	inactive	inactive
cytochrome CYP2D6	active	active
cytochrome CYP3A4	inactive	inactive
cytochrome CYP2E1	inactive	inactive

Finally, the observed redox potential of **3-cis-PtTPyP** and **4-cis-PtTPyP** (see the Supporting Information) indicates that these porphyrins can oxidize DNA and proteins via a type I mechanism by generating radical species, favored by the electron transfer process. The generation of ROS via a type I mechanism after light exposure of these cisplatin-porphyrins has been previously reported by Jornada and co-workers to induce photoinactivation of bacteria strain.^41^

## 4 Conclusions

This study assessed the photodynamic properties of novel porphyrin photosensitizers containing cisplatin-based peripheral complexes. The DNA damage and protein denaturation assays revealed that these cisplatin-porphyrins, when exposed to white light, caused either DNA base oxidation or albumin denaturation. Therefore, the type I mechanism may be involved in the process of damage induction by these photosensitizers via electron transfer directly to biomolecules, such as DNA, proteins, and possibly membranes. Collectively, our findings highlight that photodynamic activation is essential for these molecules to cause cellular damage. The cytotoxicity evaluated 24 h and 10 days after treatments showed that **3-cis-PtTPyP** was more cytotoxic for A375 tumor cells after white light exposure, although **4-cis-PtTPyP** also displayed toxicity toward this tumor cell line. Furthermore, both **3-cis-PtTPyP** and **4-cis-PtTPyP** induced significant ROS production in A375 tumor cells as well as in the HaCat keratinocytes. In addition, the clonogenic assay demonstrated that both cisplatin-porphyrins exhibited higher cytotoxicity against the A375 cell line in the dark condition when compared to the HaCat cell line, suggesting another mechanism of damage induction independent of light in this tumor cell line. *In silico* predictions of the toxicity profile and ADMET revealed that **3-cis-PtTPyP** shows better characteristics for use in photodynamic therapy. Neither compound exhibited mutagenic toxicity in the AMES test, indicating a low risk of genetic mutations. However, **4-cis-PtTPyP** was identified as a hERG II inhibitor, suggesting a potential risk of cardiotoxicity, whereas **3-cis-PtTPyP** did not display this characteristic, making it a safer alternative with regard to cardiovascular effects. These findings underscore the importance of optimizing the structure of new photosensitizers and balancing therapeutic efficacy with safety, contributing to the development of photodynamic therapy as a viable skin cancer treatment option.

## Supplementary Material



## Notes

Additional figures for the UV–vis absorption spectra of cisplatin, **3-cis-PtTPyP**, and **4-cis-PtTPyP**; emission spectrum of the white light LED array system; electrochemical analysis of cisplatin, **3-cis-PtTPyP**, and **4-cis-PtTPyP** by cyclic voltammetry; as well as a table regarding the redox potential data of cisplatin, **3-cis-PtTPyP**, and **4-cis-PtTPyP.** (PDF)
