# Supplementary material for: Cytotoxicity and DNA Damage Ability of Isomeric meso-Tetra(cisplatin)porphyrins in Keratinocytes and Melanoma Cells Treated with Photodynamic Therapy
Source: ACS Omega. 2026 Mar 25;11(13):20126–35. doi: 10.1021/acsomega.5c08553 (PMC13063096; doi:10.1021/acsomega.5c08553)
Supplement: Supplementary file 1 [file ao5c08553_si_001.pdf]

Supporting Information

Cytotoxicity and DNA damage ability of isomeric *meso*-tetra(cisplatin)porphyrins in keratinocytes and melanoma cells treated with photodynamic therapy

Níckolas P. Peranzoni[a]; Luana B. Trentin[a]; Altevir R. Viana[a]; Bernardo A. Iglesias[b]; Erdi C. Aytar[c]; André P. Schuch[a*]

1. UV-Vis absorption spectra of cisplatin, 3-cis-PtTPyP and 4-cis-PtTPyP.

The UV-Vis electronic absorption spectroscopy of cisplatin, **3-cis-PtTPyP** and **4-cis-PtTPyP** was determined using a Shimadzu UV-2600 spectrophotometer (data interval, 1.0 nm) with DMF as solvent to avoid coordination issues induced by DMSO.

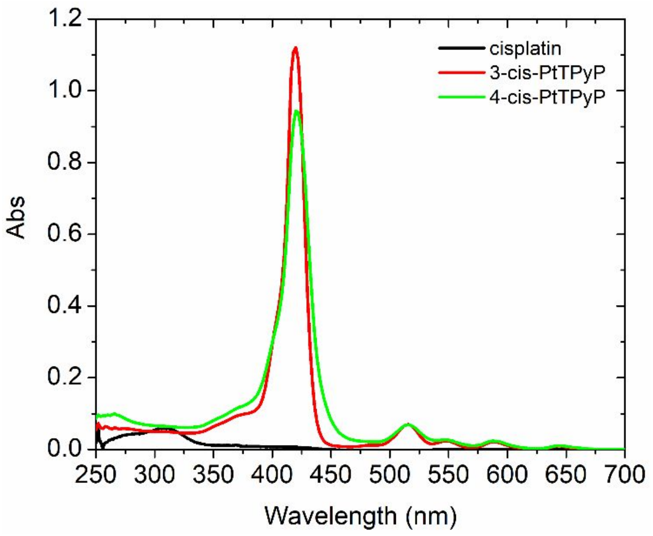

Figure S1: The UV-Vis electronic absorption spectroscopy of cisplatin, **3-cis-PtTPyP** and **4-cis-PtTPyP**.

The absorption UV-Vis spectra of the studied derivatives demonstrate that free cisplatin presents an absorption band in the UV region (around 310 nm), showing a metal-ligand charge transfer (MLCT) nature. On the other hand, tetra-cationic porphyrins **3-cis-PtTPyP** and **4-cis-PtTPyP** present absorption peaks between 400 and 450 nm, called Soret band, followed by less intense absorption between 500 and 700 nm, called Q band.

2. Emission spectrum of white light lamp.

A homemade white-light LED array system (Philps LEDs) was used in this work. The emission range is between 380-680 nm, with a more intense emission peak in the

blue region. DNA samples and cells were exposed in a 5.0 cm distance from the white light LED array system (irradiance of 50 mW/cm^2), resulting in 270 J/cm^2 dose for a period of 90 min.

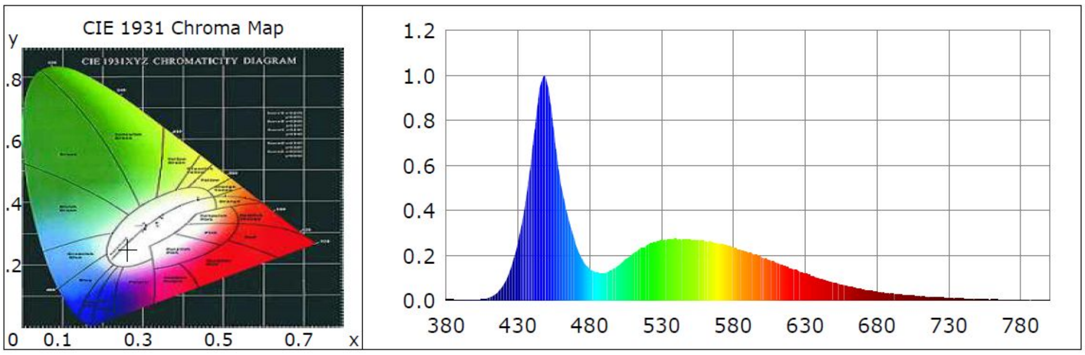

Figure S2: Emission spectrum of white-light LED lamp used in this study.

3. Electrochemical analysis by cyclic voltammetry.

Electrochemical analysis by cyclic voltammetry was performed with a potentiostat/galvanostat AutoLab Eco Chemie PGSTAT 128 N system at room temperature and under an argon atmosphere in a dry DMF solution. Electrochemical grade tetrabutylammonium hexafluorophosphate (0.1 M $TBAPF_6$) was used as a supporting electrolyte. The electrochemical cell comprises three components: a glassy carbon electrode (working electrode; 5.0 mm, Metrohm), a Pt auxiliary electrode, and a Pt *pseudo*-reference electrode. To monitor the reference electrode, the Fc/Fc+ redox couple pair ($E_{1/2}$ = 0.44 V) was used as an internal reference.

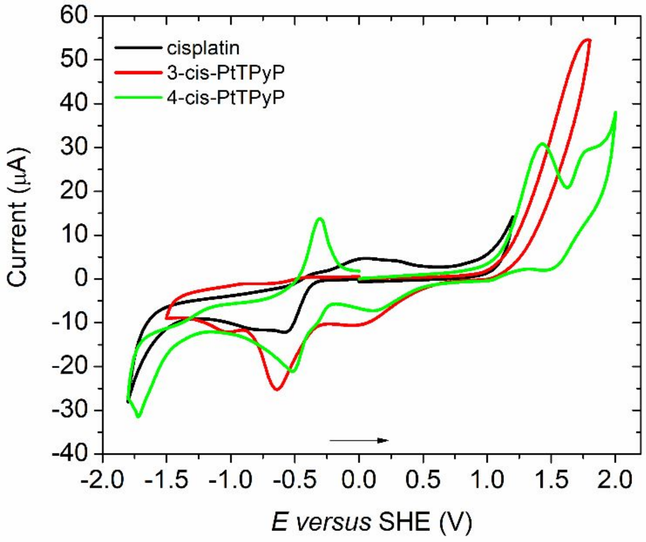

Figure S3: Electrochemical analysis of cisplatin, **3-cis-PtTPyP** and **4-cis-PtTPyP** by cyclic voltammetry in dry DMF solution, using $TBAPF_6$ as supporting electrolyte and scan rate at 100 mV/s.

Table S1. Redox potential data of derivatives, in DMF (*E versus* SHE).

Compound	E_{red1} (V)[a]	E_{red2} (V)[a]	E_{ox1} (V)[b]	E_{ox2} (V)[b]	E_{HOMO} (eV)[c]	E_{LUMO} (eV)[d]	ΔE[e]
**cisplatin**	–0.57	-----	+0.05	-----	–4.45	–3.83	0.62
**3-cis-PtTPyP**	–0.64	–1.04	+1.74	-----	–6.14	–3.76	2.38
**4-cis-PtTPyP**	–0.41*	–1.72	+1.42	+1.76	–5.82	–3.99	1.83

[a]E_{pc} = cathodic peak;

[b]E_{pa} = anodic peak;

*$E_{1/2} = E_{pa} + E_{pc} / 2$;

[c]E_{HOMO} = –[4.4 + first E_{ox} (*versus* SHE)]eV;

[d]E_{LUMO} = –[4.4 + first E_{red} (*versus* SHE)]eV;

[e]$\Delta E = E_{LUMO} - E_{HOMO}$

The studied derivatives show redox potential in the range of -2.00 to +2.00 V. In the cathodic (reduction) region, two reduction peaks can be observed for each porphyrin, which can be attributed to the formation of mono- and dianionic species stabilized in solution. In the anodic range, between +1.40 and +1.80 V, oxidation peaks were observed for both porphyrins, probably due to the formation of mono- and dicationic species, with variations in values according to the change in the coordinate to the N-pyridine position. To determine the HOMO and LUMO values, the energy equation (described in the footnote of Table S1) was used considering the first oxidation redox peak (HOMO) and reduction redox peak (LUMO) (band gap), together with a correction factor according to literature (4.4) to adjust the value to energy units (eV) [https://doi.org/10.1021/ja075242e; DOI 10.1007/s11051-014-2647-0].
